# MicroRNA-432 is downregulated in osteosarcoma and inhibits cell proliferation and invasion by directly targeting metastasis-associated in colon cancer-1

**DOI:** 10.3892/etm.2022.11402

**Published:** 2022-05-30

**Authors:** Dengkun Lv, Zhen Zhen, Defa Huang

Exp Ther Med 17:919–926, 2019; DOI: 10.3892/etm.2018.7029

Following the publication of this paper, it was drawn to the Editors’ attention by a concerned reader that the western blotting data shown in Figs. 3D, 4B and 5A, and certain of the cell migration and invasion assay data shown in Figs. 2C and 5C were strikingly similar to data appearing in different form in other articles by different authors. Owing to the fact that the contentious data in the above article had already been published elsewhere, or were already under consideration for publication, prior to its submission to *Experimental and Therapeutic Medicine,* the Editor has decided that this paper should be retracted from the Journal. After having been in contact with the authors, they agreed with the decision to retract the paper. The Editor apologizes to the readership for any inconvenience caused.

